# P53 codon 72 polymorphism, human papillomavirus infection, and their interaction to oral carcinoma susceptibility

**DOI:** 10.1186/s12863-015-0235-7

**Published:** 2015-06-30

**Authors:** Jun Hou, Ying Gu, Wei Hou, Song Wu, Yin Lou, Wenyu Yang, Ling Zhu, Yukun Hu, Ming Sun, Haowei Xue

**Affiliations:** Department of Stomatology, the First Affiliated Hospital of the Anhui Medical University, Hefei, Anhui 230032 China; Department of General Dentistry, School of Dental Medicine, Stony Brook University, Stony Brook, NY 11794 USA; Department of Preventive Medicine, School of Medicine, Stony Brook University, Stony Brook, NY 11794 USA; Department of Applied Mathematics Statistics, College of Engineering and Applied Sciences, Stony Brook University, Stony Brook, NY 11794 USA; Department of Orthopaedic Surgery, the Second Affiliated Hospital of the Anhui Medical University, Hefei, Anhui 230601 China; Department of Stomatology, the Second Affiliated Hospital of the Anhui Medical University, Hefei, Anhui 23061 China

**Keywords:** P53 codon 72, Human papillomavirus, Oral cancer, Polymorphism, Susceptibility, Meta-analysis

## Abstract

**Background:**

Tumor suppressor gene p53 plays an important role in the maintenance of the genomic integrity, and mutation in the gene may alter an individual’s susceptibility to various carcinomas. P53 Arg72Pro or codon 72 polymorphism has been indicated to increase the risk of developing certain cancers such as bladder cancer and cervical cancer. Human papillomavirus (HPV) infection has been shown as a risk factor for certain cancers such as cervical cancer and oral cancer as well, and the HPV oncoprotein E6 may induce the degradation of p53 function. However, the association between p53 Arg72Pro polymorphism and the risk of oral cancer with HPV infection remains inconclusive. Therefore, this meta-analysis involving 5,614 participants was performed to investigate the relations among the p53 Arg72Pro polymorphism, HPV infection, and the risk of developing oral cancer.

**Results:**

A search of the literature by PubMed, Embase, Web of Science, and China National Knowledge Infrastructure databases was conducted to identify studies based on the inclusion and exclusion criteria. Odds ratios with 95 % confidence intervals were combined using a random-effect model or a fixed-effect model. The current study was conducted with 13 studies consisting of 2,413 cases and 3,201 controls. Neither overall analysis nor stratified analyses detected any obvious evidence of association between p53 Arg72Pro polymorphism and oral cancer susceptibility in all genetic models. However, a significant association between p53 Arg72Pro polymorphism and the risk of oral cancer with HPV infection was detected in the Arg/Arg vs. Arg/Pro + Pro/Pro model.

**Conclusion:**

In the current meta-analysis which used the quantitative data synthesis for the first time, our study demonstrated that p53 Arg72Pro polymorphism together with HPV infection might jointly alter an individual’s susceptibility to the risk of oral cancer. Our results suggested that p53 Arg72Pro polymorphism may partly contribute to the pathogenesis of oral cancer development.

## Background

The incident rate for oral cancer has been increasing recently. Research studies have suggested that smoking, alcohol consumption, and betel quid chewing are risk factors that predispose individuals to oral cancer [1–3]. Nevertheless, only some smokers, alcohol users, and betel quid users develop oral cancer, which indicated that it can be a multifactorial process associated with various risk factors for oral cancer development. These exogenous carcinogens may induce a defective DNA damage response, which may alter the expression of tumor suppressor genes apoptosis or may result in genomic instability [4, 5]. Accumulative evidence indicates that individual susceptibility to oral cancer also depends on genetic predisposition and viral infection [6, 7]. Therefore, both environmental and genetic factors may play an important role in the process of oral cancer development.

Many published studies have reported that oral carcinoma susceptibility is associated with gene polymorphism. In recent years, much attention has been focused on the p53 codon 72 Arg/Pro polymorphism. The p53 tumor suppressor gene is located at human chromosome 17 and encoding a 53-kDa nuclear phosphoprotein which plays a crucial role in cell cycle regulation, maintenance of genomic integrity, apoptosis, and challenge of environmental insults [8, 9]. Mutant p53 codon 72 may allow cells with environment-associated damaged DNA to enter the cell cycle, leading to the development of tumors [10, 11]. In fact, there have been extensive research studies demonstrated that p53 Arg72Pro polymorphism played an important role in developing cervical cancer in HPV-positive patients. Odds of developing cervical cancer was significantly higher with the p53 Arg allele in HPV associated cervical cancer. This association was not detected in HPV-negative patients [12]. In addition, the association between p53 Arg72Pro polymorphism and oral cancer has been investigated, however, the results were inconsistent.

HPV infection have been proved as an independent risk factor for the development of oral cancer [13, 14]. The viral E6 protein, which encoded by two high risk HPV types named HPV-16 and HPV-18, was testified to bind and inactivate the human p53 gene product, and marking it for destruction by the ubiquitin proteasome pathway [15–17]. Storey et al. suggested that the p53 Arg72Pro polymorphism plays a part in the development of HPV-associated cancer in 1998 for the first time [18]. Since then, researchers have investigated the combined influence of the Arg72Pro polymorphism and HPV infection in the risk of developing oral cancer, but the results remained inconclusive [19–21].

Therefore, whether or not p53 Arg72Pro polymorphism can increase the risk of oral cancer with HPV infection remains unclear. Based on the above reasons, we conducted this evidence-based quantitative meta-analysis to investigate the relationship between p53 polymorphisms and the risk of HPV-related oral cancer.

## Methods

### Search strategy

Relevant articles were searched using combinations of search terms “oral”, “oral cavity”, “buccal”, “oropharynx”, “oral cancer”, “oral carcinoma”, “oral squamous cell carcinoma”, “ameloblastoma”, “P53”, “TP53”, “Arg72Pro”, “HPV”, “human papillomavirus”, “polymorphism”, “susceptibility”, and “gene variants”, in PubMed, Embase, Web of Science, and China National Knowledge Infrastructure databases, focusing on articles which were published from their earliest entry points to April 2014.

### Inclusion and exclusion criteria

The following inclusion criteria were used for the selection of literature for meta-analysis: (1) published in English; (2) examined case–control studies investigating the association between HPV infection, Arg72Pro polymorphism, and the risk of oral cancer; (3) definite histopathologic diagnosis; and (4) genotype distribution in controls must be in Hardy-Weinberg equilibrium (HWE). Major exclusion criteria included: (1) the unpublished reports and abstracts; (2) when duplicated studies published by the same author, only the most recent publication study was chosen.

### Data extraction

All the eligible articles were independently reviewed and extracted by two reviewers (YL and WY) according to the selection criteria listed above. Disagreement was resolved by the third independent investigator (JH). The following data were extracted from the each study: the first author, year of publication, country, ethnicity, genotyping methods, source of the controls, and genotype numbers from the cases and controls.

### Statistical analysis

The STATA version 11.0 software (Stata Corporation, College Station, TX) was used to conduct the statistical analyses. The combined odds ratio (OR) with a corresponding 95 % confidence interval (CI) was estimated to evaluate the relationship among p53 Arg72Pro polymorphisms, HPV infection, and the risk of oral cancer. For control groups, the goodness-of-fit test (Chi-square test or Fisher exact test) was used to test the deviations from HWE. The following statistical models were used in the meta-analysis: the allelic model (Arg72 allele vs. Pro72 allele), the codominant model (homozygote comparison: Arg/Arg vs. Pro/Pro), the dominant model (Arg/Arg + Arg/Pro vs. Pro/Pro), and the recessive model (Arg/Arg vs. Arg/Pro + Pro/Pro). Statistics Q and I^2^ statistic were evaluated to investigate the between-study heterogeneity [22, 23]. Either the random-effect model or the fixed-effect model was used to calculate the pooled effect estimate either in the presence or in the absence of heterogeneity, respectively [24, 25]. Additionally, the Begg’s funnel plot and the Egger’s test were used to estimate the publication bias (*p* < 0.05 was considered statistically significant) [26, 27].

## Results

### Studies characteristics

**Fig. 1:**
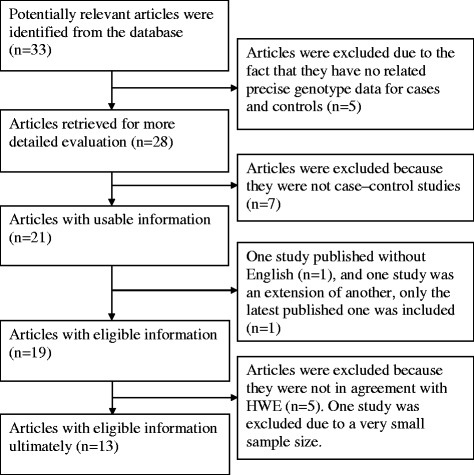
Flow diagram of the publication selection process

As shown in Fig. 1, 13 studies with a total of 5,614 participants met the inclusion and exclusion criteria [19–21, 28–37]. The characteristics of these included articles were summarized in Table 1. All the related distribution of p53 codon 72 polymorphism genotype frequencies in cases and controls were summarized in Table 2.

**Table 1:** Main characteristics of studies included in the meta-analysis

Study	Country	Ethnicity	Control source	Genotyping Methods	Sample size (case/control)
Patel KR et al. [28]	India	Asian	healthy	PCR-RFLP	79/110
Wang Z et al. [18]	USA	Caucasian	healthy	PCR-RFLP	320/321
Ji X et al. [19]	USA	Caucasian	healthy	PCR-RFLP	188/342
Kuroda Y et al. [29]	Japanese	Asian	Hospital	PCR-RFLP	100/271
Kitkumthorn N et al. [30]	Thailand	Asian	healthy	PCR-RFLP	78/94
Chen X et al. [31]	USA	Caucasian	healthy	PCR-RFLP	326/349
Zemleduch T et al. [32]	Caucasian	Caucasian	healthy	PCR-RFLP	123/300
Ihsan R et al. [33]	India	Asian	healthy	PCR-RFLP	116/278
Tu HF et al. [34]	Taiwan	Asian	healthy	DNA sequence	189/116
Summersgill KF et al. [20]	USA	Caucasian	Hospital	PCR-CTPP	190/308
Misra C et al. [35]	India	Asian	Hospital	PCR-RFLP	308/342
Lin YC et al. [36]	Taiwan	Asian	unknown	PCR-RFLP	297/280
Saini R et al. [37]	Malaysia	Asian	healthy	PCR-CTPP	99/90

**Table 2:** Distribution of p53 codon 72 genotypes among oral cancer in cases and controls

	Cases (n)			Controls (n)			
First author	Arg/Arg	Arg/Pro	Pro/Pro	Arg/Arg	Arg/Pro	Pro/Pro	P-value of HWE in controls
Patel KR et al. [28]	32	29	18	30	58	22	0.528
Wang Z et al. [18]	43	41	15	24	15	2	0.860
Ji X et al. [19]	103	74	11	179	140	23	0.532
Kuroda Y et al. [29]	41	44	15	109	117	45	0.159
Kitkumthorn N et al. [30]	35	40	3	27	47	20	0.957
Chen X et al. [31]	183	121	22	181	144	24	0.518
Zemleduch T et al. [32]	55	52	16	176	104	20	0.389
Ihsan R et al. [33]	30	63	23	63	143	72	0.619
Tu HF et al. [34]	53	106	30	41	60	15	0.337
Summersgill KF et al. [20]	102	70	18	168	112	28	0.144
Misra C et al. [35]	87	155	66	85	159	98	0.203
Lin YC et al. [36]	96	155	46	72	152	56	0.135
Saini R et al. [37]	22	40	37	28	39	23	0.215

HWE: Hardy–Weinberg equilibrium

### Meta-analysis results

#### The association between p53 Arg72Pro polymorphism and the risk of oral cancer in total population

**Fig. 2:**
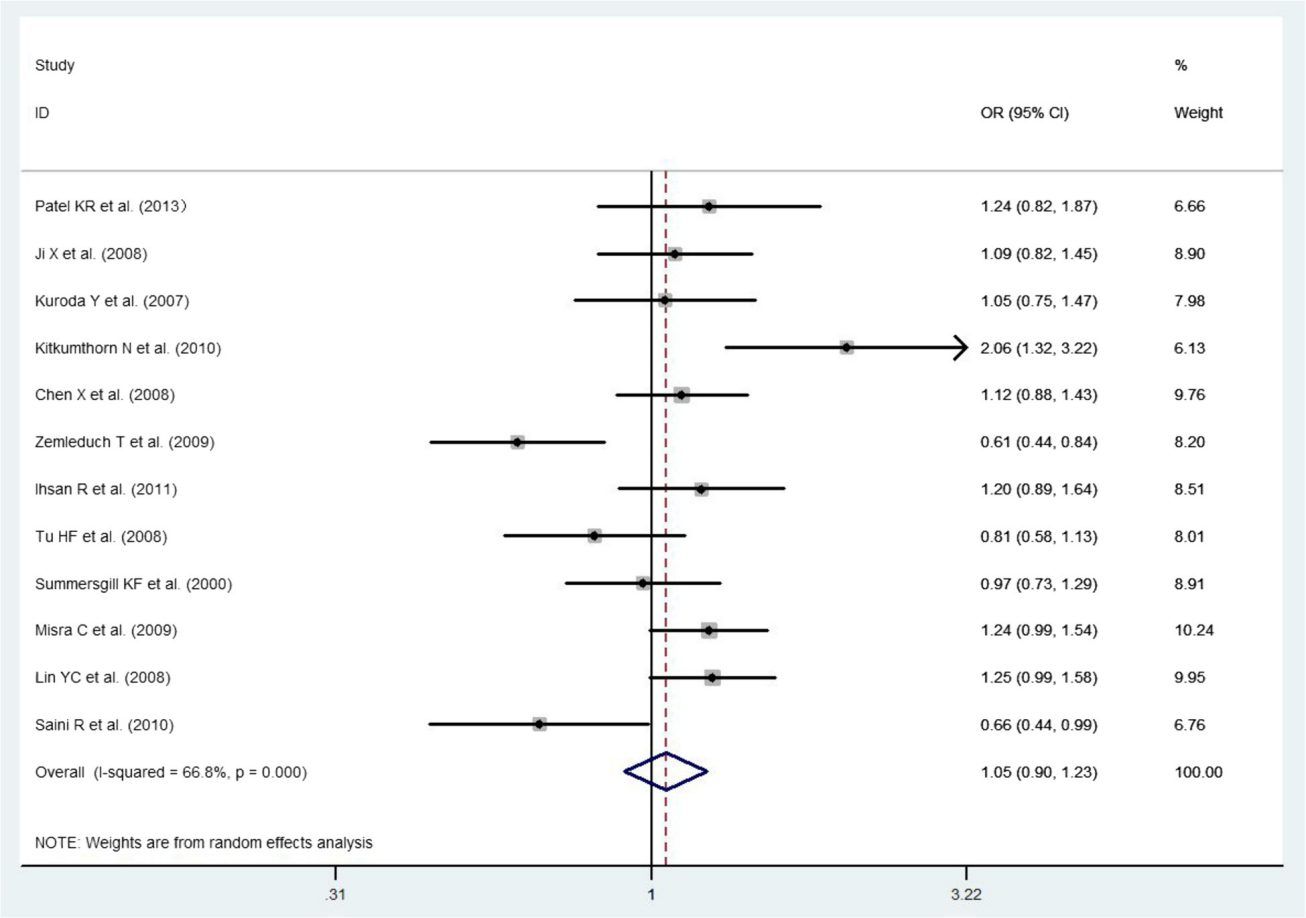
The association between p53 Arg72Pro polymorphism and the risk of oral cancer in total population (Arg72 allele vs. Pro72 allele)

**Fig. 3:**
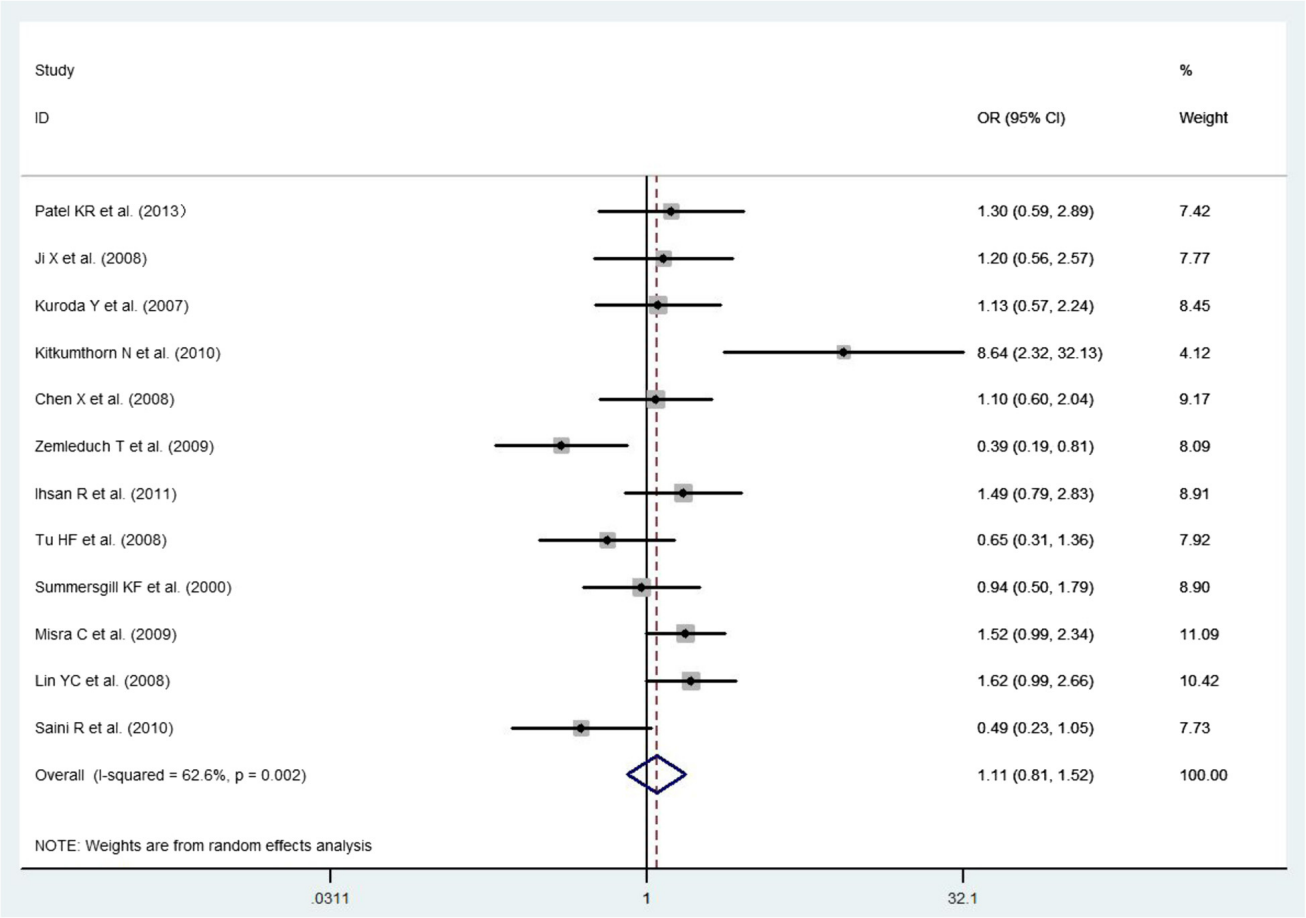
The association between p53 Arg72Pro polymorphism and the risk of oral cancer in total population (Arg/Arg vs. Pro/Pro)

**Fig. 4:**
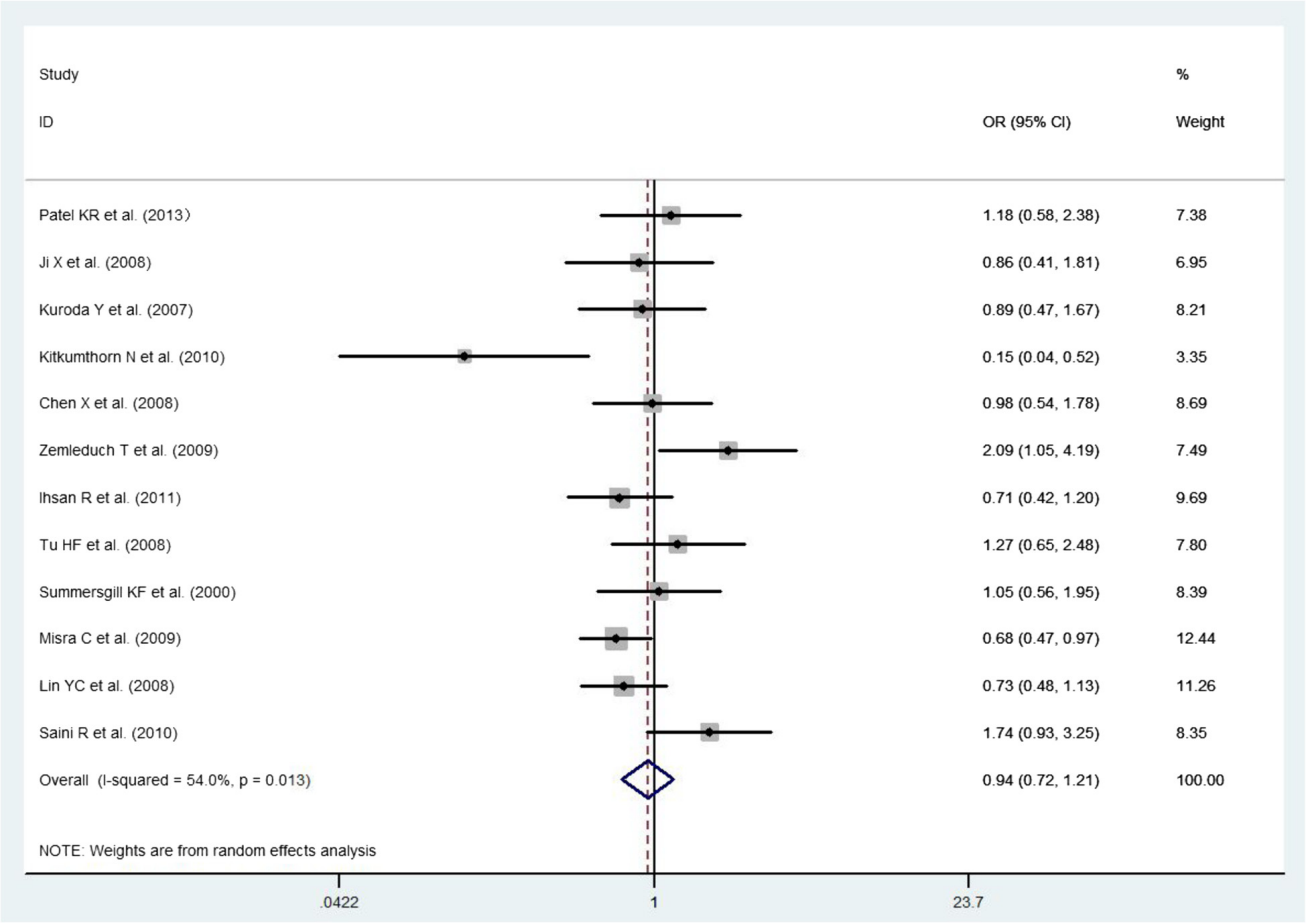
The association between p53 Arg72Pro polymorphism and the risk of oral cancer in total population (Arg/Arg + Arg/Pro)

**Fig. 5:**
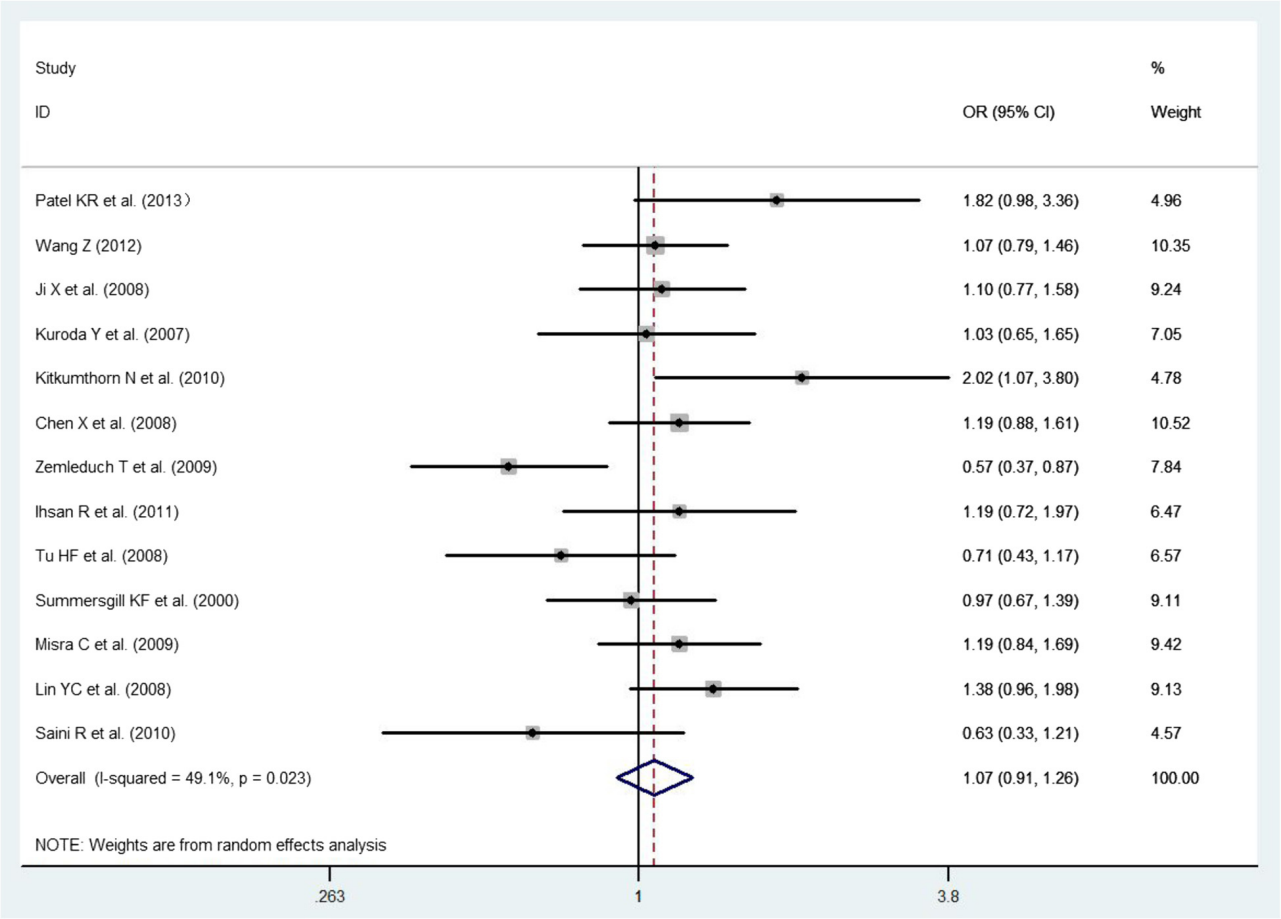
The association between p53 Arg72Pro polymorphism and the risk of oral cancer in total population (Arg/Arg vs. Arg/Pro + Pro/Pro)

A total of 13 studies were included in the meta-analysis to examine the association between p53 Arg72Pro polymorphism and the risk of oral cancer. There was no evidence of a significant association in any genetic model (Arg72 allele vs. Pro72 allele: OR = 1.05, 95 % CI: 0.90- 1.23; Arg/Arg vs. Pro/Pro: OR = 1.11, 95 % CI: 0.81- 1.52; Pro/Pro vs. Arg/Arg + Arg/Pro: OR = 0.94, 95 % CI: 0.72- 1.21; Arg/Arg vs. Arg/Pro + Pro/Pro: OR = 1.07, 95 % CI: 0.91- 1.26; all p values >0.05; Figs. 2, 3, 4 and 5, Table 3). However, significant heterogeneity across the studies was present in four genetic models (*P* = 0.000, 0.002, 0.013, 0.023 for the allelic genetic model, the homozygote comparison model, the dominant model and the recessive model, respectively Table 3).

**Table 3:** Association, test heterogeneity and publication bias for p53 Arg72Pro polymorphism and the risk of oral cancer

Comparison	Number of studies	Sample size (case/control)	Test of association				Test of heterogeneity			Publication bias	
			OR	95 %CI	P value	Model	Q	P value	I^2^	P value (Begg’s)	P value (Egger’s)
Arg72 allele vs. Pro72 allele											
Total	12	2,093/2,880	1.054	0.905-1.228	0.500	R	33.16	0.000	66.8 %	0.300	0.202
Caucasian	4	827/1,299	0.933	0.722-1.206	0.597	R	9.92	0.019	69.7 %	0.221	0.175
Asian	8	1,266/1,581	1.128	0.934-1.362	0.210	R	19.40	0.007	63.9 %	0.902	0.717
Arg/Arg vs. Pro/Pro											
Total	12	2,093/2,880	1.109	0.807-1.524	0.523	R	29.39	0.002	62.6 %	0.360	0.415
Caucasian	4	827/1,299	0.870	0.621-1.218	0.416	F	6.04	0.110	50.3 %	0.462	0.312
Asian	8	1,266/1,581	1.277	0.860-1.898	0.226	R	19.36	0.007	63.8 %	0.536	0.914
Arg/Arg + Arg/Pro vs. Pro/Pro											
Total	12	2,093/2,880	0.936	0.723-1.211	0.613	R	23.90	0.013	54.0 %	0.161	0.423
Caucasian	4	827/1,299	1.142	0.823-1.583	0.427	F	3.81	0.283	21.2 %	0.806	0.451
Asian	8	1,266/1,581	0.846	0.613-1.169	0.312	R	17.10	0.017	59.1 %	0.711	0.990
Arg/Arg vs. Arg/Pro + Pro/Pro											
Total	13	2,413/3,201	1.069	0.907-1.259	0.426	R	23.56	0.023	49.1 %	0.511	0.302
Caucasian	5	1,147/1,620	0.975	0.777-1.224	0.828	R	8.54	0.074	53.2 %	0.060	0.054
Asian	8	1,266/1,581	1.161	0.917-1.471	0.215	R	13.19	0.068	46.9 %	0.902	0.883
HPV infection	5	396/213	0.677	0.478-0.959	0.027	F	0.93	0.920	0.0 %	0.462	0.400

Model Abbreviations: R = random-effect; F = fixed-effect

#### The association between p53 Arg72Pro polymorphism and the risk of oral cancer in a specific population

In order to determine the major cause for the heterogeneity, a stratified analysis of the specific populations was performed. Eight studies were conducted in Asian populations and five studies were conducted in Caucasian populations. No significant association between the risk of oral cancer and p53 codon 72 polymorphism was detected in the Asian and the Caucasian groups in any genetic model (Table 3). Significant heterogeneity was detected in both groups in all genetic models, except for Pro/Pro vs. Arg/Arg + Arg/Pro in the Caucasian group (Table 3).

#### The association between combined effect of p53 Arg72Pro polymorphism with HPV infection and the risk of oral cancer in total population

A total of five studies, including 396 cases and 213 controls, were included to evaluate the relations among HPV, p53 Arg72Pro polymorphism, and oral cancer susceptibility. The result showed that the association of HPV with p53 Arg72Pro variant genotypes displayed a statistical significance on oral cancer risk in the Arg/Arg vs. Pro carriers (Arg/Pro + Pro/Pro) model (OR: 0.68, 95 % CI: 0.48- 0.96, *p* = 0.028) (Fig. 2, Table 3). There was no significant heterogeneity among these studies (Q = 0.93, I^2^ = 0.0 %, *P* = 0.92; Table 3).

### Publication bias

Begg’s funnel plots seemed to be approximately symmetrical in all meta-analyses (data not shown). Additionally, Egger’s tests did not reveal any obvious evidence of publication bias either (Table 3).

## Discussion

Since the identification of the p53 codon 72 polymorphism, many studies have been devoted to explore the genetic effect of p53 Arg72Pro polymorphism on susceptibility of oral cancer. However, the evidence regarding the role of single nucleotide polymorphism of p53 Arg72Pro gene as a genetic marker for the risk of oral cancer is inconsistent. This prompted us to undertake the present meta-analysis to explore a more robust estimate of the relationship between p53 Arg72Pro genetic variant and the oral cancer susceptibility. In this study, we found that individuals who have genetic variants (Arg/Pro genotype or Pro/Pro genotype) may not have induced modification of oral cancer risk compared with those who carry wild-type genotype (Arg/Arg genotype). Same SNP may play different roles in the development of cancer in different ethnic populations. Therefore, the relation of p53 Arg72Pro polymorphism with oral cancer susceptibility might be affected by the different ethnic groups. Nevertheless, neither Arg/Arg genotype individuals nor Pro carriers have a significant association with oral carcinoma in the Asian group or the Caucasian group.

HPV belongs to a large virus family, the PAPOVA virus family. There are nearly a hundred types of HPV discovered in human [38]. In this family, some of the members are known to be high-risk oncogenic HPV type, such as HPV-16, HPV-18, HPV-33, and HPV-58. Through encoding oncogenic protein E6, high-risk HPV types inhibit p53 cell cycle tumor suppressor. The viral E6 protein has a powerful binding affinity for p53 protein resulting in its ubiquitination and destruction, thereby inducing degradation of p53 function and cell cycle out of control [15]. Therefore, p53 gene may have some interaction with HPV infection in susceptibility to HPV-associated oral cancer. Some investigators have found that joint action of the p53 codon 72 polymorphism with HPV is associated with the risk of oral cancer [19, 20, 30], but different conclusions were obtained by other investigators [21, 36]. Considering the above mentioned conflicting conclusions, a subgroup analysis of interaction of p53 gene polymorphism with HPV infection on oral cancer susceptibility was performed. Our study demonstrated a significant interaction between HPV infection and p53 Arg72Pro polymorphism on the risk of developing oral cancer in p53 Arg/Arg genotype carriers compared with p53 72Pro carriers.

The small sample size is a major limitation in this study. There were only five research articles investigating the interaction between the infection with HPV and p53 codon 72 polymorphism on the risk of oral carcinoma. Thus, additional studies with larger sample size are needed to further evaluate the impact of HPV infection and p53 Arg72Pro polymorphism on HPV-associated oral cancer susceptibility.

## Conclusion

For the first time, the current study provided the quantitatively synthesized estimates for the effect of interaction between HPV infection and p53 Arg72Pro polymorphism on the risk of developing oral cancer. This combined effect might together alter an individual’s susceptibility to oral cancer. Our results suggested that p53 Arg72Pro polymorphism may partly contribute to the pathogenesis of oral cancer development. Further well-designed studies with reference to the interactions of gene-gene and gene-environment on p53 codon 72 polymorphism to oral carcinoma susceptibility are required.

## Abbreviations

HPV: Human papillomavirus
OR: Odds ratio
CI: Confidence interval
HWE: Hardy-Weinberg equilibrium
